# Assessing Long‐Term Changes in Endometrial Morphology and Functionality in Dairy Cows With Metritis

**DOI:** 10.1111/rda.70206

**Published:** 2026-04-15

**Authors:** M. H. Rashid, A. Martelo Pereira, F. N. S. Pereira, M. C. Oliveira Costa, E. F. Santos, L. Christensen, J. A. Sexton, M. Ciccarelli, L. B. Williams, M. Binelli, M. S. Waqas, E. B. de Oliveira, C. C. Figueiredo

**Affiliations:** ^1^ Department of Veterinary Clinical Sciences Field Disease Investigation Unit, Washington State University Pullman Washington USA; ^2^ Department of Animal, Veterinary and Food Sciences University of Idaho Moscow Idaho USA; ^3^ Department of Veterinary Microbiology and Pathology, Washington Animal Disease Diagnostic Laboratory Washington State University Pullman Washington USA; ^4^ Department of Animal Sciences University of Florida Gainesville Florida USA; ^5^ Zoetis Inc Parsippany New Jersey USA

**Keywords:** fertility, MultiOmics, uterus

## Abstract

This study evaluated the long‐term impact of metritis on endometrial morphology and functionality in dairy cows. Postpartum Holstein cows (*n* = 107) were enrolled in a cohort study at the University of Idaho Dairy Center. Metritis was diagnosed using a Metricheck device within 12 days in milk (DIM) based on the presence of fetid, watery, reddish‐brown vaginal discharge (VD). Cows diagnosed with metritis (MET; *n* = 9) were treated with ceftiofur crystalline free acid and paired with cows without metritis (NoMET; *n* = 9) of similar parity and DIM. Ovulation was synchronized using a Double Ovsynch protocol starting at 35 DIM, and ovulation was confirmed by ultrasonography. To eliminate confounding effects of semen or conceptus‐derived signals, cows were not inseminated. Six days after ovulation (68–75 DIM), endometrial biopsy and cytology samples were collected. Endometrial morphology was assessed by histological evaluation of haematoxylin and eosin–stained sections. Endometrial transcriptomic profiles were determined by RNA sequencing from tissues collected via biopsy, and uterine fluid collected during cytology was analysed using metabolomic profiling. Differential gene expression was assessed using DESeq2, and metabolomic differences were evaluated using partial least squares discriminant analysis and PERMANOVA. Cows with metritis exhibited a numerically greater prevalence of periglandular fibrosis (63%; 95% Exact Binomial Confidence Interval [CI] = 25–92) compared with NoMET cows (44%; 95% CI = 14–79). Transcriptomic analysis identified 17 differentially expressed genes (FDR ≤ 0.10; |log2 fold change| > 1), including upregulation of genes associated with tissue repair in MET cows. No differences were detected in uterine fluid metabolomic profiles between groups. In conclusion, metritis was associated with persistent alterations in endometrial morphology and gene expression that were detectable months after clinical resolution, although these changes were not reflected in the uterine fluid metabolome.

## 1 Introduction

Reproductive efficiency is central to the sustainability of dairy herds, as timely establishment of pregnancy is required to maintain a positive income over feed cost. Because the cost of milk production increases as lactation progresses, delays in pregnancy are associated with higher maintenance costs and substantial economic losses, particularly in high‐producing cows (Groenendaal et al. 2004; Meadows et al. 2005; De Vries 2006). Uterine diseases further compromise herd sustainability by prolonging time to pregnancy and reducing milk yield. Metritis consistently decreases pregnancy rates during the first three postpartum inseminations (Merenda et al. 2021; Ribeiro et al. 2013; Santos et al. 2010), increases pregnancy loss (22% vs. 12%; Ribeiro et al. 2016), and extends median days to pregnancy by approximately 30 days (Figueiredo et al. 2021). In addition, metritis is associated with significant reductions in milk yield (Figueiredo et al. 2024; Wittrock et al. 2011). Collectively, these effects contribute to an estimated US$1.2 billion in annual losses to the U.S. dairy industry (Pérez‐Báez et al. 2021), underscoring the need to better understand the mechanisms underlying its long‐term effects on fertility.

Multiple biological pathways have been implicated in the subfertility associated with metritis, including persistent uterine inflammation, tissue damage, and prolonged alterations in ovarian function. Cows with metritis have a higher prevalence of purulent vaginal discharge, a condition associated with reduced fertility (Lima et al. 2014; Merenda et al. 2021; Ojeda‐Rojas et al. 2025). Structural and functional uterine alterations persisting into mid‐lactation have also been reported, including reduced endometrial gland development and epithelial proliferation (Sellmer Ramos et al. 2023), increased fibrosis and adenomyosis (Sellmer Ramos et al. 2025), elevated epithelial inflammation scores (Caldeira et al. 2025; Moraes et al. 2025), and upregulation of inflammatory and oxidative stress pathways (Caldeira et al. 2025; Silva et al. 2024). In parallel, metritis has been associated with delayed resumption of estrous cyclicity (Bruinjé et al. 2024; Figueiredo et al. 2024), a recognized contributor to subfertility (Gonzalez et al. 2023).

Together, these long‐term alterations in endometrial morphology and ovarian function may create a uterine environment that is unfavourable for embryo development and implantation. Supporting this concept, embryos collected 15 days after fertilization from cows previously affected by metritis and retained fetal membranes were smaller and secreted less interferon‐τ than those from healthy cows (Ribeiro et al. 2016). Because preimplantation embryos rely exclusively on histotroph produced by endometrial glands, alterations in the uterine fluid metabolome near the time of breeding may contribute to reduced fertility but remain poorly characterized. Therefore, we hypothesized that cows previously affected by metritis exhibit long‐term differences in endometrial morphology and function, including the uterine fluid metabolome, compared with cows without metritis. The objective of this study was to evaluate these long‐term uterine changes associated with metritis, with particular emphasis on the uterine fluid metabolome.

## 2 Materials and Methods

### 2.1 Ethics Statement

All procedures performed in this study were approved by the Washington State University Institutional Animal Care and Use Committee (IACUC; ASAF 7206).

### 2.2 Study Population and Metritis Diagnosis

This was a prospective cohort study conducted at the University of Idaho Dairy Center (Herd size: approximately 120 lactating cows) located in Moscow, ID, between October 2023 and October 2024. A total of 107 Holstein dairy cows were examined for vaginal discharge (VD) characteristics using a Metricheck device at 4, 6, 8, 10 and 12 days in milk (DIM). The VD was scored using a 5‐point scale described for dairy cows (Figueiredo et al. 2024). Cows with fetid, watery and red‐brown discharge were deemed affected by metritis (MET, *n* = 12; Primiparous, *n* = 3; Multiparous, *n* = 9) and subsequently treated with 2 doses of ceftiofur crystalline‐free acid (6.6 mg/kg of body weight; Excede, Zoetis Inc., USA), as per manufacturer indication. Cows that had clear, mucopurulent or purulent VD were considered not affected by metritis (NoMET, *n* = 12; Primiparous, *n* = 2; Multiparous, *n* = 10), and were paired with MET cows based on similar DIM and parity.

### 2.3 Purulent Vaginal Discharge Diagnosis, Synchronization of Ovulation and Sample Collection

Presence of purulent vaginal discharge (PVD; mucopurulent and purulent VD) was assessed in all enrolled cows at 35 DIM using the Metricheck device and scored as described previously (Dubuc et al. 2010).

To control the time to ovulation, all cows at 35 DIM were enrolled in a synchronization of ovulation protocol (Double‐Ovsynch) as previously described (Souza et al. 2008). Briefly, all cows received GnRH (100 μg of gonadorelin diacetate tetrahydrate, Zoetis, Parsippany, NJ) at 35 DIM, followed by an injection of PGF_2α_ (25 μg of dinoprost tromethamine, Zoetis, Parsippany, NJ) at 42 DIM, and GnRH at 45 DIM. The second portion of the protocol was conducted similarly: GnRH was administered at 52 DIM, PGF_2α_ at 59 DIM, and GnRH at 62 DIM. Because a portion of cows are likely to ovulate prior to the last GnRH injection, transrectal ovarian ultrasonography was conducted daily following the last PGF_2α_ injection (at 59 DIM) to confirm ovulation day. Ovulation was considered as the disappearance of dominant follicle (> 8 mm), and day of ovulation was considered as study day 0 (occurred between 62 and 67 DIM). The ovulation side (i.e., right or left ovary) was recorded to further guide sample collection. To avoid confounding effects of semen, extender, or conceptus‐derived signals, cows were not inseminated.

On Day 6 (occurred between 68 and 75 DIM), endometrial samples for metabolomic characterization of uterine fluid, and endometrial morphology and transcriptomics were collected using cytological brushes and biopsy forceps as previously described (Silva et al. 2021, 2023; Simintiras et al. 2019). Briefly, the perineal area of cows was disinfected using a 90% alcohol solution and paper towels. Double‐guarded cytology brush (MAI Animal Health, Elmwood, WI) and cytological samples were collected from the base of the uterine horn ipsilateral to the ovulation side. The presence of the corpus luteum was confirmed ultrasonographically prior to sampling. Immediately after collection, the cytobrush was transferred to a cryogenic tube (Nunc, Roskilde, Denmark) containing 10 mL of sterile water (Fisher Scientific, Waltham, MA). The cryotube containing the cytology brush was vortexed for 2 min to transfer contents from the brush into the sterile water. The brush was discarded and the cryotube was frozen in liquid nitrogen. Following cytology procedures, endometrial biopsy samples (ipsilateral to ovulation and corpus luteum) were collected via Jackson uterine forceps (Jorgensen Laboratories, Loveland, CO). The collected tissue (approximately 30 mm) was divided into two similarly sized pieces, one reserved for histopathology and the other for transcriptomic analysis. Tissues for histopathology were placed in a tube containing Bouin's solution for 2 min and later transferred to tubes containing a 90% alcohol solution for storage. Tissues for transcriptomic analysis were placed in a cryotube containing RNA*later* Stabilization Solution (Thermo Fisher Scientific, Waltham, MA) and frozen in nitrogen shortly after. Within 4 h of collection and freezing in nitrogen, samples were stored in –80°C until processing time.

### 2.4 Metabolomic, Transcriptomic and Histopathologic Analyses

Endometrial samples collected by cytology were analysed at the West Coast Metabolomics Center (CA, USA). Primary metabolites were quantified using gas chromatography time‐of‐flight mass spectrometry (GC‐TOF MS) with an ALEX‐CIS system, and complex lipids were analysed by ultra‐high‐performance liquid chromatography coupled with quadrupole time‐of‐flight mass spectrometry. Complex lipid annotation was performed using in‐house mass‐to‐charge ratio and retention time libraries and by mass spectral matching against the NIST and MoNA databases. Laboratory procedures and data processing for quality control were performed as previously described (Fiehn et al. 2008; Fiehn 2016). Briefly, to minimize analytical variability for GC‐TOF MS, guard‐column trimming, automated liner exchanges and fatty acid methyl ester (FAME) internal standards supported retention‐index stability and contamination control, while strict BinBase filters ensured only high‐confidence metabolites contributed to final data. For complex lipids, internal standards were similarly monitored for consistent peak height and retention time, with peak identification curated using MS‐DIAL and NIST/MoNA spectral libraries. Across both platforms, pooled quality control samples monitored run‐sequence drift and informed normalization (mTIC or iTIC), ensuring the final metabolite and lipid values remained directly comparable and interpretable.

For transcriptomic analysis, one of the portions from endometrial tissue collected via biopsy was processed by IDSeq Laboratories (CA, USA). Libraries were prepared by mRNA enrichment using oligo(dT)‐attached magnetic beads, followed by mRNA fragmentation, cDNA synthesis using random N6 primers, and PCR amplification. Single‐stranded circular DNA libraries were generated and sequenced on the DNBSEQ platform (150 bp paired‐end reads) after quality control. Sequencing reads were aligned to the reference genome using HISAT2 (Kim et al. 2015), followed by transcript‐level alignment with Bowtie2 (Langmead and Salzberg 2012) and gene expression quantification using RSEM (Li and Dewey 2011).

Endometrial histopathology was conducted at the Washington Animal Disease Diagnostic Laboratory by a board‐certified pathologist. Samples were cleared with xylene, embedded in paraffin, sectioned, and stained with haematoxylin and eosin. Sections were evaluated for gland density (glands per 10× field), presence of fibrosis, and periglandular spindle cell layers, as previously described (Bonnett et al. 1991). In addition, endometrial biopsies were independently evaluated by a board‐certified theriogenologist and graded using the Kenney–Doig system (Snider et al. 2011): Grade I (normal or mild focal inflammation/fibrosis), Grade IIA (mild to moderate inflammation and/or multifocal fibrosis), Grade IIB (moderate inflammation and/or multifocal to diffuse fibrosis) and Grade III (severe inflammation and/or diffuse fibrosis).

### 2.5 Sample Size and Statistical Analysis

Sample size calculation strategies using metabolomic data are scarce; therefore, we opted to use sample size calculation strategies developed for transcriptomic data, considering previous endometrial transcriptomic studies conducted in Angus cattle (Adhikari et al. 2022; Alfattah et al. 2024). Using *ssizeRNA* R package, a minimum of 6 cows/group was necessary to detect log2 fold‐change (log2FC) difference of more than 2 in at least 5% of genes with 80% power while false discovery rate‐corrected *p* < 0.05 (Bi and Liu 2019). To account for losses of enrolled cows due to culling or limitations related to sampling, we enrolled 12 cows per group.

All the analyses in the current study were done in R (R Core Team 2024) via RStudio interface (Posit team 2024). Primary metabolites and complex lipids analysis were done using the same methods. Overall differences between the groups were observed using partial least squares discriminant analysis (PLS‐DA) after autoscaling the data in the *mixOmics* R package (Rohart et al. 2017). A permutational multivariate ANOVA (PERMANOVA) with 9999 permutations was done via the *vegan* R package to formally test the group level differences (Oksanen et al. 2023). Subsequently, differences in the abundance of specific metabolites were compared via *t*‐test followed by correction for false discovery rate (FDR) using the Benjamini‐Hochberg method (Benjamini and Hochberg 1995). Significance threshold was defined as FDR < 0.05.

For differential gene expression, the gene expression matrix was loaded in RStudio and analysed via *DESeq2* R package (Love et al. 2014). The raw count matrix was fitted to a DESeqDataSetFromMatrix object, with Parity (Primiparous and Multiparous) included as a random effect and Group (MET and NoMET) as the main effect. The significance threshold was set at FDR < 0.1 and |log2FC| > 1. Principal component analysis (PCA) followed by PERMANOVA was performed to explore the overall trend in gene expression data. No inferential statistics were performed for histopathology or PVD due to few data points, as preliminary power calculation indicated that a minimum of 120 endometrial biopsies/group would be necessary to detect a 9‐percentage point difference in risk of periglandular fibrosis between groups; hence, only descriptive statistics are reported.

## 3 Results

Of the 24 cows enrolled (12 per group), four failed to ovulate following synchronization and were excluded (MET = 2; NoMET = 2). One additional MET cow was excluded because endometrial biopsy and cytology could not be performed due to cervical size. Endometrial biopsy was also not obtained from two cows (one per group) for the same reason. Consequently, sample sizes differed by analysis, with 8 MET and 9 NoMET cows included in transcriptomic and histopathologic analyses, and 9 cows per group included in metabolomic analyses.

### 3.1 Metabolome of the Uterine Fluid on Day 6 After Ovulation

**FIGURE 1:**
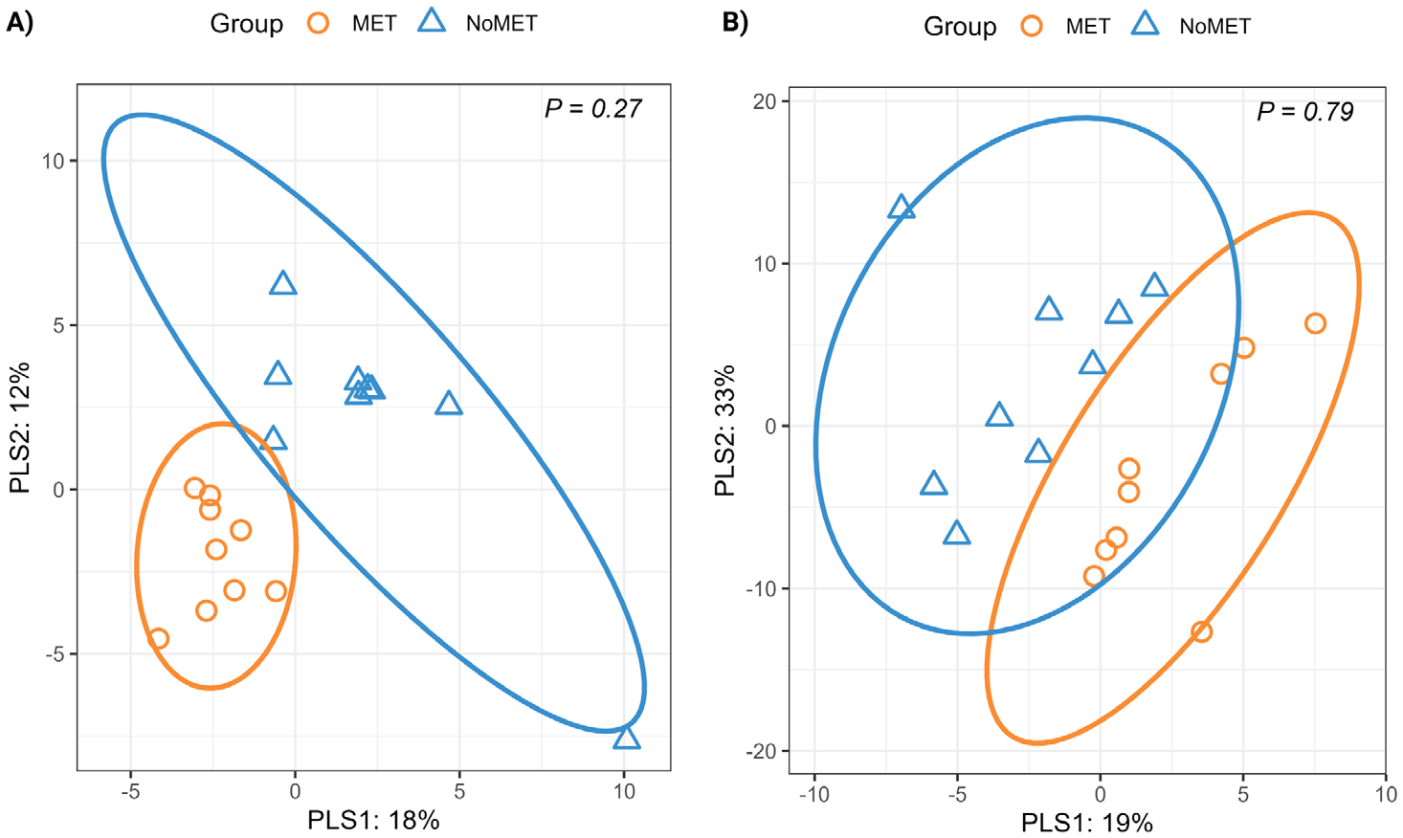
Partial Least Squares‐Discriminant Analysis of primary metabolites (A) and complex lipids (B) in cows with a history of metritis (MET, *n* = 9) or no metritis (NoMET, *n* = 9). *p*‐values represent the results of Permutational multivariate ANOVA.

A total of 240 metabolites were identified, but only 114 were annotated (Supplemental Table S1; https://doi.org/10.6084/m9.figshare.30768365). Only annotated metabolites were used for downstream analysis. The PLS‐DA analysis of the primary metabolites showed close approximation among MET group samples but greater variance among NoMET group samples. No differences were observed between the two groups on PERMANOVA (Figure 1A, *p* = 0.27). A total of 542 complex lipids were identified; however, only 193 of these lipids could be annotated (Table S2; https://doi.org/10.6084/m9.figshare.30768365). The PLS‐DA analysis showed an overlap between MET and NoMET groups, which was supported by PERMANOVA findings (Figure 1B, *p* = 0.79). No differences were observed in the abundance of primary metabolites and complex lipids (FDR > 0.05).

### 3.2 Transcriptome of the Endometrium on Day 6 After Ovulation

**FIGURE 2:**
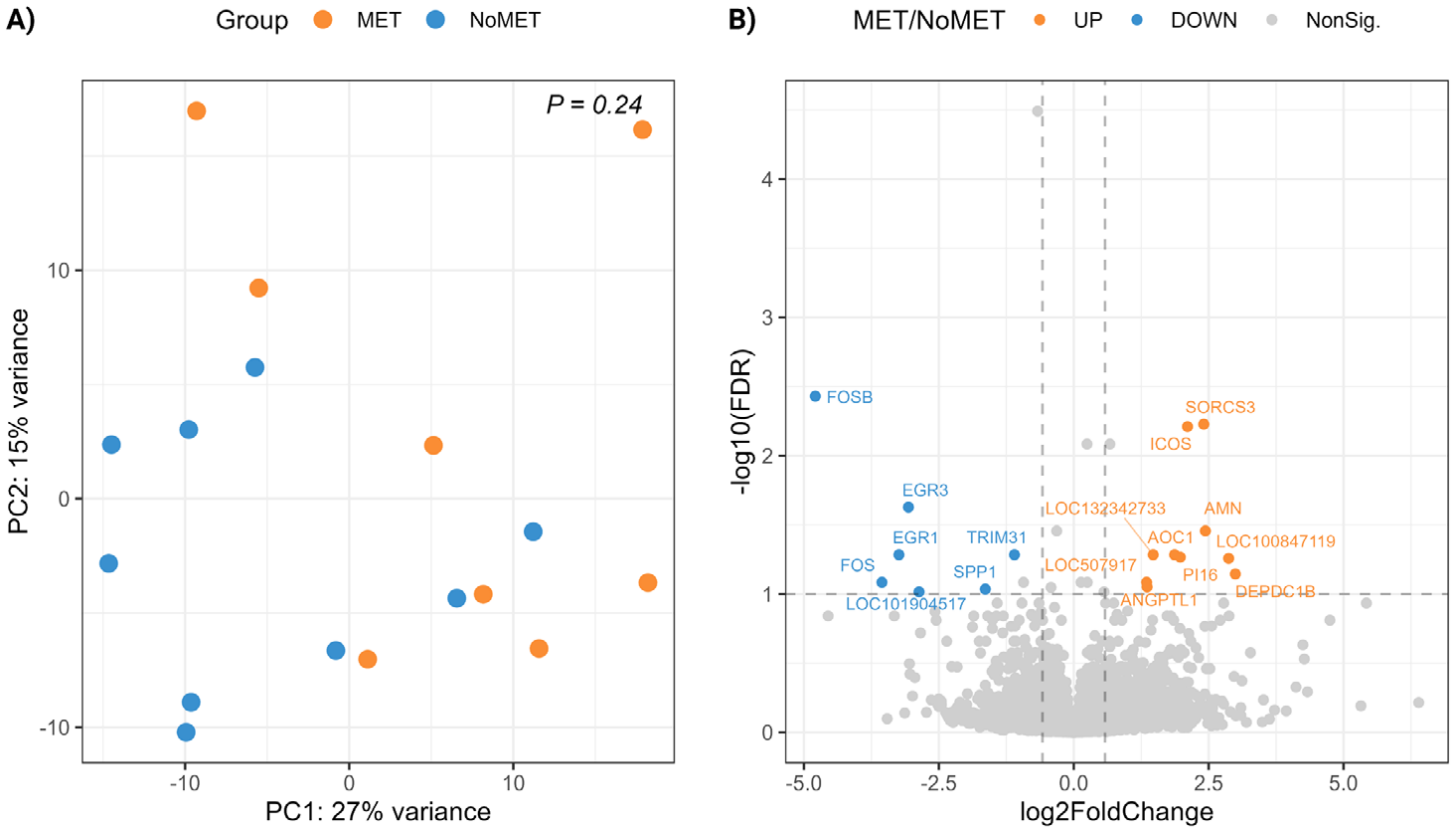
(A) Principal Component Analysis of gene expression data, and (B) volcano plot summarizing the differentially expressed genes between the endometrium of cows with a history of metritis (MET, *n* = 8) and no metritis (NoMET, *n* = 9).

For gene expression analysis, a mean count of 16,550,098 reads/sample was obtained with a mean count of 16,516,912 reads/sample for MET and 16,579,598 reads/sample for NoMET (Table S3; https://doi.org/10.6084/m9.figshare.30768365). Dimensionality reduction was done via PCA and while PC1 and PC2 explained 42% variance (27% and 15%, respectively), this was not explained by our groups. The lack of clustering among MET and NoMET samples was also supported by PERMANOVA results (*p* = 0.24; Figure 2). The gene expression analysis via *DESeq2* showed that 17 genes were differentially expressed (FDR < 0.1, |log2FC| > 1) between MET and NoMET groups. Compared with NoMET, 10 genes were upregulated and 7 were downregulated in MET (Table 1). Due to an insufficient number of differentially expressed genes, functional enrichment analysis could not be performed.

**TABLE 1:** List of differentially expressed genes between cows with a history of metritis (MET, *n* = 8) or no metritis (NoMET; *n* = 9; reference group).

Gene symbol	Gene name	Log2FC	FDR
*Upregulated in MET*			
SORCS3	Sortilin related VPS10 domain containing receptor 3	2.41	< 0.01
ICOS	Inducible T cell costimulator	2.11	< 0.01
AMN	Amnion associated transmembrane protein	2.44	0.03
LOC132342733	Zinc finger protein 160‐like	1.47	0.05
AOC1	Amine oxidase copper containing 1	1.87	0.05
PI16	Peptidase inhibitor 16	1.97	0.05
LOC100847119	Immunoglobulin lambda variable 3–25	2.87	0.06
DEPDC1B	DEP domain containing 1B	2.99	0.07
LOC507917	MHC class I heavy chain	1.35	0.08
ANGPTL1	Angiopoietin like 1	1.36	0.09
*Downregulated in MET*			
FOSB	FosB proto‐oncogene, AP‐1 transcription factor subunit	−4.79	< 0.01
EGR3	Early growth response 3	−3.06	0.02
EGR1	Early growth response 1	−3.24	0.05
TRIM31	Tripartite motif containing 31	−1.09	0.05
FOS	Fos proto‐oncogene, AP‐1 transcription factor subunit	−3.55	0.08
SPP1	Secreted phosphoprotein 1	−1.64	0.09
LOC101904517	ATP‐binding cassette sub‐family C member 4‐like	−2.87	0.10

### 3.3 Prevalence of PVD at 35 DIM and Endometrial Morphology on Day 6 After Ovulation

**FIGURE 3:**
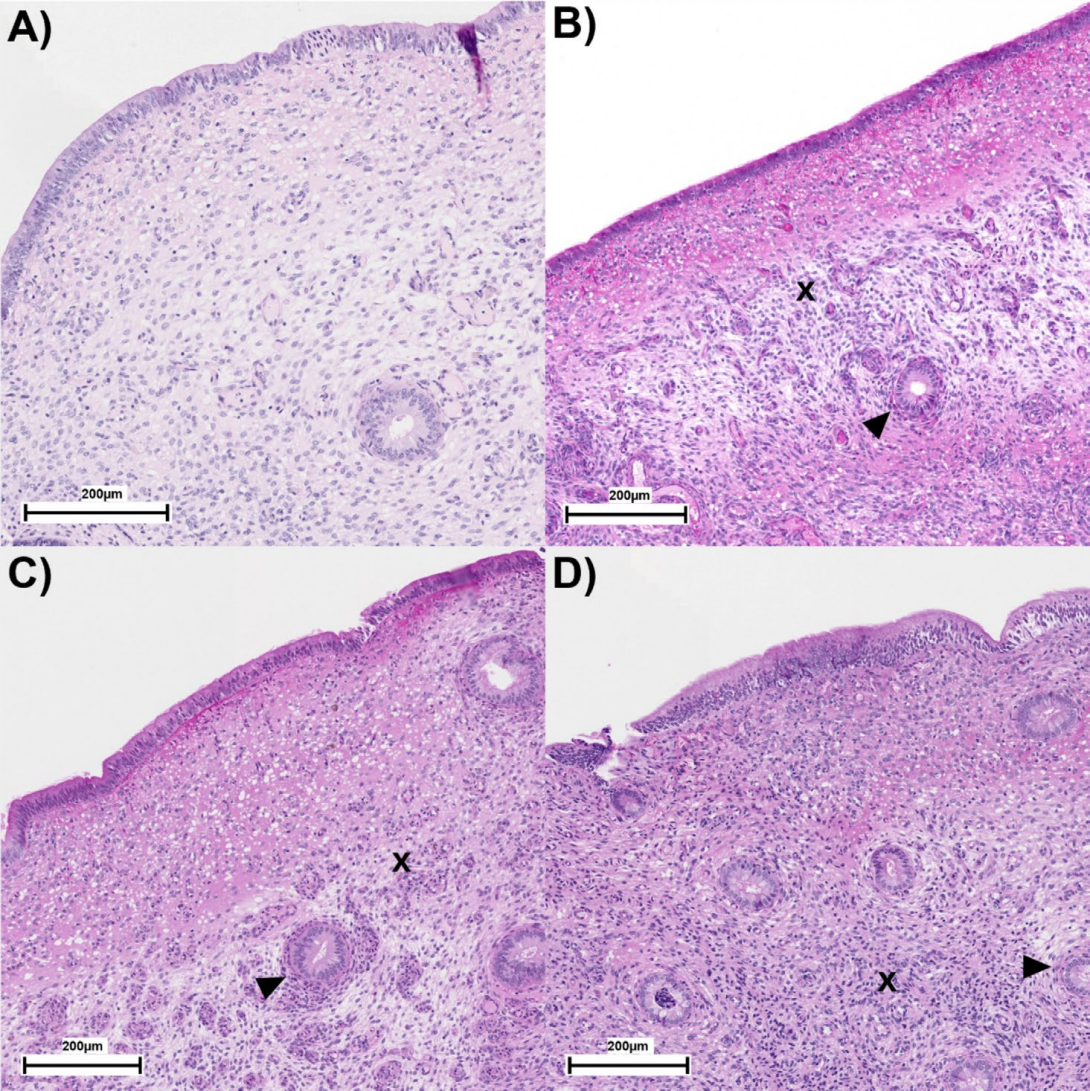
Endometrial biopsies graded using the Kenney‐Doig grading system. Grade I (Panel A): Normal endometrium or mild, focal inflammation or fibrosis. Grade IIA (Panel B): Mild to moderate inflammation and/or multifocal fibrosis. Grade IIB (Panel C): Moderate inflammation and/or multifocal to diffuse fibrosis. Grade III (Panel D): Severe inflammation and/or diffuse fibrosis. Arrowheads represent peri‐glandular fibrosis, while x represents increased cellular density (diffused inflammation).

At 35 DIM, a numerically greater proportion of MET cows were affected by PVD compared with NoMET cows (56 [5/9 cows] vs. 11% [1/9 cows]). Histopathological examination of endometrial biopsies on Day 6 after ovulation showed that a numerically greater proportion of cows in MET had periglandular fibrosis (63%; 95% Exact Binomial Confidence Interval = 25–92) as compared to NoMET (44%; 95% Exact Binomial Confidence Interval = 14–79) cows. Yet, a similar number of glands was observed per 10× field in MET and NoMET (Mean ± Standard Deviation: 13 ± 9 and 15 ± 12 glands, respectively). The assessment of endometrial biopsies based on Kenney‐Doig grading system highlighted that a numerically greater proportion of MET cows present severe endometrial degeneration and inflammation compared with NoMET cows. The distribution of MET and NoMET cows across endometrial grading groups is as follows: Grade I: 13 [1/8] vs. 22% [2/9]; Grade IIA: 25 [2/8] vs. 33% [3/9]; Grade IIB: 13 [1/8] vs. 33% [3/9]; and Grade III: 50 [4/8] vs. 11% [1/9]. Representative morphology slides are presented in Figure 3.

## 4 Discussion

The objective of this study was to evaluate long‐term changes in endometrial morphology and function, with particular emphasis on the uterine fluid metabolome, associated with metritis. We observed persistent differences in endometrial morphology and gene expression in cows previously affected by metritis, characterized by higher endometrial degeneration scores and differential expression of genes linked to inflammation and tissue repair. In contrast, no differences were detected in the uterine fluid metabolome between cows with and without metritis.

Consistent with expectations, metritis was associated with alterations in endometrial architecture that were also reflected at the transcriptomic level. These findings align with previous reports documenting persistent endometrial inflammation and structural abnormalities from early postpartum through mid‐lactation (Sellmer Ramos et al. 2023, 2025; Silva et al. 2024; Caldeira et al. 2025; Moraes et al. 2025). Although different methodologies were used to assess endometrial architecture, increased inflammation has been a consistent feature of metritis. The persistence of these alterations at approximately 60 DIM, a time corresponding to insemination in most U.S. dairy herds, may partially explain the reduced fertility observed in affected cows. Supporting this interpretation, endometrial biopsy grading systems, widely applied in mares, have been associated with marked reductions in conception and pregnancy maintenance as biopsy scores increase (Snider et al. 2011).

Despite clear differences in endometrial morphology and transcriptome, the absence of detectable differences in uterine fluid metabolome was unexpected. Few studies have examined the uterine fluid metabolome beyond the acute phase of metritis, but similar findings have been reported at 30 DIM (Caldeira et al. 2025). In that study, metritis per se was not associated with changes in the uterine proteome; however, differences emerged when cows were stratified by vaginal discharge characteristics, with proteins related to immune function differentially regulated between cows with purulent versus clear discharge. As in that study, a greater proportion of cows with metritis in the present study subsequently developed purulent vaginal discharge, suggesting persistent immune activation. It is therefore plausible that immunological alterations associated with metritis either did not translate into measurable changes in uterine fluid metabolites or occurred at a magnitude below the detection power of the current sample size.

Nonetheless, transcriptomic analysis identified metritis‐associated differential expression of genes involved in immune regulation (e.g., ICOS) and tissue repair or angiogenesis (e.g., ANGPTL1 and DEPDC1B). Upregulation of ICOS may reflect a compensatory response to persistent inflammation through enhanced T‐cell differentiation (Hosking et al. 2025), whereas increased expression of ANGPTL1 and DEPDC1B may indicate ongoing attempts at vascular remodelling and restoration of endometrial function (Xue et al. 2025; Zhang et al. 2024). In contrast, cows without a history of metritis exhibited greater expression of FOS, FOSB, EGR1 and EGR3, genes associated with hormone‐responsive endometrial proliferation, suggesting a more intact and functionally responsive endometrium (Gellersen and Brosens 2014; Kim et al. 2014).

Additional studies assessing the uterine fluid metabolome in dairy cows from different geographic regions, using diverse sampling strategies and analytical platforms, are needed. This study was conducted in a single herd in the northwestern United States, and although the findings align with reports from Midwestern herds, they may not be fully generalizable to other regions. Furthermore, although a priori power calculations were performed, they were based solely on endometrial gene expression. Tools for sample size calculation for metabolome have not been extensively developed, underscoring the need for improved power estimation methods for metabolomic studies.

Long‐term differences in endometrial morphology and function associated with uterine repair were observed. However, the absence of differences in primary metabolites and complex lipids, together with the limited number of differentially expressed genes, suggests that histopathological alterations between MET and NoMET cows may not be reflected in the uterine metabolomic profile. These findings highlight the need for studies focused on improving metritis prevention, as cows that achieved clinical resolution following antimicrobial treatment still exhibited persistent uterine alterations.

## 5 Conclusion

We investigated the long‐term morphological, metabolomic, and gene expression changes associated with the history of metritis. While the morphological differences (qualitatively assessed), including periglandular fibrosis and shifts in endometrial gene expression were associated with metritis, the same did not translate at the metabolomic level (primary and complex lipids). We concluded that cows with a history of metritis have long‐term differences in endometrial morphology and function, which may complement the complex underlying mechanisms of subfertility observed for that group.

## Author Contributions

Conceptualization: C.C.F., M.C., M.B., M.S.W.; Methodology: C.C.F., M.C., M.B., M.S.W., L.C., J.A.S., L.B.W.; Investigation/Data Collection: M.H.R., A.M.P., F.N.S.P., M.C.O., E.F.S., M.S.W., C.C.F.; Formal Analysis: M.H.R., L.B., M.C., C.C.F.; Data Curation: M.H.R., C.C.F.; Writing – Original Draft: M.H.R.; Writing – Review and Editing: C.C.F., M.C., L.B.W., E.B.D.O.; Funding Acquisition: C.C.F.

## Funding

This work was supported by the U.S. Department of Agriculture (7005594).

## Conflicts of Interest

The authors declare no conflicts of interest.

## Supporting information


**Table S1:** Abundance level of primary metabolites in uterine luminal fluid in MET and NoMET cows. The metabolites were identified via gas chromatography with time‐of‐flight mass spectrometry (GC‐TOF‐MS).
**Table S2:** Abundance level of complex lipids in uterine luminal fluid in MET and NoMET cows. The metabolites were identified via Ultra‐High‐Performance Liquid Chromatography with a Quadrupole Time‐of‐Flight Mass Spectrometer (UHPLC‐QTOF‐MS).
**Table S3:** Gene expression matrix (raw reads) of endometrium in MET and NoMET cows.

## Data Availability

The data that support the findings of this study are openly available in Figshare at https://doi.org/10.6084/m9.figshare.30768365, reference number 30768365.

## References

[rda70206-bib-0001] Adhikari, B. , C. N. Lee , V. S. Khadka , et al. 2022. “RNA‐Sequencing Based Analysis of Bovine Endometrium During the Maternal Recognition of Pregnancy.” BMC Genomics 23, no. 1: 494. 10.1186/s12864-022-08720-4. 35799127 PMC9264496

[rda70206-bib-0002] Alfattah, M. A. , C. N. Correia , J. A. Browne , et al. 2024. “Transcriptomics Analysis of the Bovine Endometrium During the Perioestrus Period.” PLoS One 19, no. 3: e0301005. 10.1371/journal.pone.0301005. 38547106 PMC10977793

[rda70206-bib-0003] Benjamini, Y. , and Y. Hochberg . 1995. “Controlling the False Discovery Rate: A Practical and Powerful Approach to Multiple Testing.” Journal of the Royal Statistical Society. Series B, Statistical Methodology 57, no. 1: 289–300.

[rda70206-bib-0004] Bi, R. , and P. Liu . 2019. ssizeRNA: Sample Size Calculation for RNA‐Seq Experimental Design [Computer Software]. https://CRAN.R‐project.org/package=ssizeRNA.

[rda70206-bib-0005] Bonnett, B. N. , S. W. Martin , V. P. Gannon , R. B. Miller , and W. G. Etherington . 1991. “Endometrial Biopsy in Holstein‐Friesian Dairy Cows. I. Technique, Histological Criteria, and Results.” Canadian Journal of Veterinary Research 55: 155–161. 1884295 PMC1263436

[rda70206-bib-0006] Bruinjé, T. C. , E. I. Morrison , E. S. Ribeiro , D. L. Renaud , and S. J. LeBlanc . 2024. “Associations of Inflammatory and Reproductive Tract Disorders Postpartum With Pregnancy and Early Pregnancy Loss in Dairy Cows.” Journal of Dairy Science 107: 1630–1644. 10.3168/jds.2023-23976. 37820756

[rda70206-bib-0007] Caldeira, M. O. , J. G. N. Moraes , T. T. Nguyen , et al. 2025. “Impact of Metritis and Systemic Antibiotic Treatment on the Biology and Morphology of the Bovine Uterus at One Month Postpartum.” Biology of Reproduction 113: 1132–1154. 10.1093/biolre/ioaf146. 40613309

[rda70206-bib-0008] De Vries, A. 2006. “Economic Value of Pregnancy in Dairy Cattle.” Journal of Dairy Science 89: 3876–3885. 10.3168/jds.S0022-0302(06)72430-4. 16960063

[rda70206-bib-0009] Dubuc, J. , T. F. Duffield , K. E. Leslie , J. S. Walton , and S. J. LeBlanc . 2010. “Definitions and Diagnosis of Postpartum Endometritis in Dairy Cows.” Journal of Dairy Science 93: 5225–5233. 10.3168/jds.2010-3428. 20965337

[rda70206-bib-0010] Fiehn, O. 2016. “Metabolomics by Gas Chromatography–Mass Spectrometry: Combined Targeted and Untargeted Profiling.” Current Protocols in Molecular Biology 114: 30.4.1–30.4.32. 10.1002/0471142727.mb3004s114. PMC4829120 27038389

[rda70206-bib-0011] Fiehn, O. , G. Wohlgemuth , M. Scholz , et al. 2008. “Quality Control for Plant Metabolomics: Reporting MSI‐Compliant Studies.” Plant Journal 53: 691–704. 10.1111/j.1365-313X.2007.03387.x. 18269577

[rda70206-bib-0012] Figueiredo, C. C. , S. Casaro , F. Cunha , et al. 2024. “Evaluating Differences in Milk Production, Reproductive Performance, and Survival Associated With Vaginal Discharge Characteristics and Fever in Postpartum Dairy Cows.” Journal of Dairy Science 107, no. 8: 6079–6089. 10.3168/jds.2023-23905. 38580147

[rda70206-bib-0013] Figueiredo, C. C. , V. R. Merenda , E. B. de Oliveira , et al. 2021. “Failure of Clinical Cure in Dairy Cows Treated for Metritis Is Associated With Reduced Productive and Reproductive Performance.” Journal of Dairy Science 104: 7056–7070. 10.3168/jds.2020-19661. 33741169

[rda70206-bib-0014] Gellersen, B. , and J. J. Brosens . 2014. “Cyclic Decidualization of the Human Endometrium in Reproductive Health and Failure.” Endocrine Reviews 35: 851–905. 10.1210/er.2014-1045. 25141152

[rda70206-bib-0015] Gonzalez, T. D. , L. Factor , A. Mirzaei , et al. 2023. “Targeted Reproductive Management for Lactating Holstein Cows: Reducing the Reliance on Exogenous Reproductive Hormones.” Journal of Dairy Science 106: 5788–5804. 10.3168/jds.2022-22666. 37349211

[rda70206-bib-0016] Groenendaal, H. , D. T. Galligan , and H. A. Mulder . 2004. “An Economic Spreadsheet Model to Determine Optimal Breeding and Replacement Decisions for Dairy Cattle.” Journal of Dairy Science 87: 2146–2157. 10.3168/jds.S0022-0302(04)73335-3. 15328228

[rda70206-bib-0017] Hosking, S. L. , L. M. Moldenhauer , H. M. Tran , et al. 2025. “Treg cells promote decidual vascular remodeling and modulate uterine NK cells in pregnant mice.” JCI Insight 10, no. 2: e169836. 10.1172/jci.insight.169836. PMC11790030 39656539

[rda70206-bib-0018] Kim, D. , B. Langmead , and S. L. Salzberg . 2015. “HISAT: A Fast Spliced Aligner With Low Memory Requirements.” Nature Methods 12, no. 4: 357–360. 10.1038/nmeth.3317. 25751142 PMC4655817

[rda70206-bib-0019] Kim, J. J. , E. C. Sefton , and S. E. Bulun . 2014. “Progesterone Receptor Action in Leiomyoma and Endometrial Cancer.” Progress in Molecular Biology and Translational Science 124: 53–85. 10.1016/B978-0-12-800095-3.00003-0. PMC3838876 20374701

[rda70206-bib-0020] Langmead, B. , and S. L. Salzberg . 2012. “Fast Gapped‐Read Alignment With Bowtie 2.” Nature Methods 9, no. 4: 357–359. 10.1038/nmeth.1923. 22388286 PMC3322381

[rda70206-bib-0021] Li, B. , and C. N. Dewey . 2011. “RSEM: Accurate Transcript Quantification From RNA‐Seq Data With or Without a Reference Genome.” BMC Bioinformatics 12: 323. 10.1186/1471-2105-12-323. 21816040 PMC3163565

[rda70206-bib-0022] Lima, F. S. , A. Vieira‐Neto , G. S. Vasconcellos , et al. 2014. “Efficacy of Ampicillin Trihydrate or Ceftiofur Hydrochloride for Treatment of Metritis and Subsequent Fertility in Dairy Cows.” Journal of Dairy Science 97: 5401–5414. 10.3168/jds.2013-7569. 24952780

[rda70206-bib-0023] Love, M. I. , W. Huber , and S. Anders . 2014. “Moderated Estimation of Fold Change and Dispersion for RNA‐Seq Data With DESeq2.” Genome Biology 15, no. 12: 550. 10.1186/s13059-014-0550-8. 25516281 PMC4302049

[rda70206-bib-0024] Meadows, C. , P. J. Rajala‐Schultz , and G. S. Frazer . 2005. “A Retrospective Study of the Association Between Postpartum Metritis and Reproductive Performance in Dairy Cows.” Theriogenology 64: 151–163. 10.1016/j.theriogenology.2004.11.007.

[rda70206-bib-0025] Merenda, V. R. , D. Lezier , A. Odetti , et al. 2021. “Effects of Metritis Treatment Strategies on Health, Behavior, Reproductive, and Productive Responses of Holstein Cows.” Journal of Dairy Science 104: 2056–2073. 10.3168/jds.2020-19076. 33309374

[rda70206-bib-0026] Moraes, J. G. N. , T. Gull , A. C. Ericsson , et al. 2025. “Evaluating Differences in Uterine Microbiome and Inflammatory Status at 1 Month Postpartum Associated With Metritis and Antibiotic Treatment.” Journal of Dairy Science 108: 13958–13980. 10.3168/jds.2025-26403. 40975288 PMC12991386

[rda70206-bib-0027] Ojeda‐Rojas, O. A. , J. Pérez‐Báez , S. Casaro , et al. 2025. “The Economic Impact of Purulent Vaginal Discharge in Dairy Herds Within a Single Lactation.” Journal of Dairy Science 108: 2710–2720. 10.3168/jds.2024-24897. 39662820

[rda70206-bib-0028] Oksanen, J. , G. Simpson , F. Blanchet , et al. 2023. vegan: Community Ecology Package. Comprehensive R Archive Network. https://github.com/vegandevs/vegan.

[rda70206-bib-0029] Pérez‐Báez, J. , T. V. Silva , C. A. Risco , et al. 2021. “The Economic Cost of Metritis in Dairy Herds.” Journal of Dairy Science 104: 3158–3168. 10.3168/jds.2020-19125. 33455790

[rda70206-bib-0030] Posit team . 2024. RStudio: Integrated Development Environment for R. www.posit.co.

[rda70206-bib-0031] R Core Team . 2024. R: A Language and Environment for Statistical Computing (Version 2024.9.0.375). R Foundation for Statistical Computing. https://www.R‐project.org.

[rda70206-bib-0032] Ribeiro, E. S. , G. Gomes , L. F. Greco , et al. 2016. “Carryover Effect of Postpartum Inflammatory Diseases on Developmental Biology and Fertility in Lactating Dairy Cows.” Journal of Dairy Science 99: 2201–2220. 10.3168/jds.2015-10337. 26723113

[rda70206-bib-0033] Ribeiro, E. S. , F. S. Lima , L. F. Greco , et al. 2013. “Prevalence of Periparturient Diseases and Effects on Fertility of Seasonally Calving Grazing Dairy Cows Supplemented With Concentrates.” Journal of Dairy Science 96: 5682–5697. 10.3168/jds.2012-6335. 23831093

[rda70206-bib-0034] Rohart, F. , B. Gautier , A. Singh , and K.‐A. L. Cao . 2017. “mixOmics: An R Package for ‘Omics Feature Selection and Multiple Data Integration.” PLoS Computational Biology 13, no. 11: e1005752. 10.1371/journal.pcbi.1005752. 29099853 PMC5687754

[rda70206-bib-0035] Santos, J. E. , R. S. Bisinotto , E. S. Ribeiro , et al. 2010. “Applying Nutrition and Physiology to Improve Reproduction in Dairy Cattle.” Society for Reproduction and Fertility. Supplement 67: 387–403. 10.5661/RDR-VII-387. 21755686

[rda70206-bib-0036] Sellmer Ramos, I. , M. O. Caldeira , S. E. Poock , J. G. N. Moraes , M. C. Lucy , and A. L. Patterson . 2025. “Adenomyosis and Fibrosis Define the Morphological Memory of the Postpartum Uterus of Dairy Cows Previously Exposed to Metritis.” JDS Communications 6: 250–255. 10.3168/jdsc.2024-0633. 40405988 PMC12094055

[rda70206-bib-0037] Sellmer Ramos, I. , J. G. N. Moraes , M. O. Caldeira , S. E. Poock , T. E. Spencer , and M. C. Lucy . 2023. “Impact of Postpartum Metritis on the Regeneration of Endometrial Glands in Dairy Cows.” JDS Communications 4: 400–405. 10.3168/jdsc.2022-0338. 37727237 PMC10505777

[rda70206-bib-0038] Silva, F. A. C. C. , G. F. da Silva , B. S. Vieira , et al. 2021. “Peri‐Estrus Ovarian, Uterine, and Hormonal Variables Determine the Uterine Luminal Fluid Metabolome in Beef Heifers.” Biology of Reproduction 105, no. 5: 1140–1153. 10.1093/biolre/ioab149. 34350935

[rda70206-bib-0039] Silva, F. A. C. C. , T. Martins , M. Sponchiado , et al. 2023. “Pre‐Estrus Progesterone Does Not Affect Post‐Estrus Luminal Metabolome in Cross‐Bred Beef Cows.” Reproduction 166, no. 2: 99–116. 10.1530/REP-22-0372. 37224090

[rda70206-bib-0040] Silva, J. C. C. , M. O. Caldeira , J. G. N. Moraes , et al. 2024. “Metritis and the Uterine Disease Microbiome Are Associated With Long‐Term Changes in the Endometrium of Dairy Cows.” Biology of Reproduction 111: 332–350. 10.1093/biolre/ioae067. 38704744 PMC12097902

[rda70206-bib-0041] Simintiras, C. A. , J. M. Sánchez , M. McDonald , T. Martins , M. Binelli , and P. Lonergan . 2019. “Biochemical Characterization of Progesterone‐Induced Alterations in Bovine Uterine Fluid Amino Acid and Carbohydrate Composition During the Conceptus Elongation Window†.” Biology of Reproduction 100, no. 3: 672–685. 10.1093/biolre/ioy234. 30388203

[rda70206-bib-0042] Snider, T. A. , C. Sepoy , and G. R. Holyoak . 2011. “Equine Endometrial Biopsy Reviewed: Observation, Interpretation, and Application of Histopathologic Data.” Theriogenology 75: 1567–1581. 10.1016/j.theriogenology.2010.12.013. 21356552

[rda70206-bib-0043] Souza, A. H. , H. Ayres , R. M. Ferreira , and M. C. Wiltbank . 2008. “A New Presynchronization System (Double‐Ovsynch) Increases Fertility at First Postpartum Timed AI in Lactating Dairy Cows.” Theriogenology 70, no. 2: 208–215. 10.1016/j.theriogenology.2008.03.014. 18468675

[rda70206-bib-0044] Wittrock, J. M. , K. L. Proudfoot , D. M. Weary , and M. A. von Keyserlingk . 2011. “Short Communication: Metritis Affects Milk Production and Cull Rate of Holstein Multiparous and Primiparous Dairy Cows Differently.” Journal of Dairy Science 94: 2408–2412. 10.3168/jds.2010-3697. 21524531

[rda70206-bib-0045] Xue, C. , Q. Chu , Q. Shi , Y. Zeng , J. Lu , and L. Li . 2025. “Wnt Signaling Pathways in Biology and Disease: Mechanisms and Therapeutic Advances.” Signal Transduction and Targeted Therapy 10, no. 1: 106. 10.1038/s41392-025-02142-w. 40180907 PMC11968978

[rda70206-bib-0046] Zhang, Y. , Y. Zhuang , J. Zhou , et al. 2024. “Effect of Estradiol After Bacterial Infection on the Wnt/β‐Catenin Pathway in Bovine Endometrium Epithelial Cells and Organoids.” Theriogenology 219: 75–85. 10.1016/j.theriogenology.2024.02.023. 38402700

